# Root inoculation with *Azotobacter chroococcum* 76A enhances tomato plants adaptation to salt stress under low N conditions

**DOI:** 10.1186/s12870-018-1411-5

**Published:** 2018-09-20

**Authors:** Michael James Van Oosten, Emilio Di Stasio, Valerio Cirillo, Silvia Silletti, Valeria Ventorino, Olimpia Pepe, Giampaolo Raimondi, Albino Maggio

**Affiliations:** Department of Agricultural Sciences, University of Naples Federico II, Via Università, 100 Portici, Italy

**Keywords:** Salinity, Micro tom, Tomato, Plant nutrition, *Azotobacter chroococcum*, Rhizobacteria

## Abstract

**Background:**

The emerging roles of rhizobacteria in improving plant nutrition and stress protection have great potential for sustainable use in saline soils. We evaluated the function of the salt-tolerant strain *Azotobacter chroococcum* 76A as stress protectant in an important horticultural crop, tomato. Specifically we hypothesized that treatment of tomato plants with *A. chroococcum* 76A could improve plant performance under salinity stress and sub-optimal nutrient regimen.

**Results:**

Inoculation of Micro Tom tomato plants with *A. chroococcum* 76A increased numerous growth parameters and also conferred protective effects under both moderate (50 mM NaCl) and severe (100 mM NaCl) salt stresses. These benefits were mostly observed under reduced nutrient regimen and were less appreciable in optimal nitrogen conditions. Therefore, the efficiency of *A. chroococcum* 76A was found to be dependent on the nutrient status of the rhizosphere. The expression profiles of *LEA* genes indicated that *A. chroococcum* 76A treated plants were more responsive to stress stimuli when compared to untreated controls. However, transcript levels of key nitrogen assimilation genes revealed that the optimal nitrogen regimen, in combination with the strain *A. chroococcum* 76A, may have saturated plant’s ability to assimilate nitrogen.

**Conclusions:**

Roots inoculation with *A. chroococcum* 76A tomato promoted tomato plant growth, stress tolerance and nutrient assimilation efficiency under moderate and severe salinity. Inoculation with beneficial bacteria such as *A. chroococcum* 76A may be an ideal solution for low-input systems, where environmental constraints and limited chemical fertilization may affect the potential yield.

**Electronic supplementary material:**

The online version of this article (10.1186/s12870-018-1411-5) contains supplementary material, which is available to authorized users.

## Background

Salinity affects more than 20% of global agricultural production and it is predicted to increase in its extent and severity in the coming decades [17]. Salinization occurs through both natural and anthropogenic processes [12]. Predictions based on unprecedented variations in rainfall and temperatures indicate that many sources of freshwater for irrigation are at severe risk for salinization [20]. It has been estimated that salinization renders 3 ha of arable land unproductive every minute [39]. Salinity has major impacts on food production and represents a growing challenge for developing sustainable agricultural systems [43, 57].

High concentrations of salt cause both ionic and osmotic stresses. Salinity induces osmotic stress by lowering the soil water potential, thus increasing the energy required for uptake of water and nutrients. Ionic stress is caused by the progressive accumulation of sodium and chloride ions in sensitive plant tissues [11, 16, 35]. Plants employ numerous strategies to survive and adapt to salt stress including control of sodium transport across the plasma and tonoplast membranes, osmotic adjustment, upregulation of ROS scavengers and ion compartmentalization in the vacuole [30, 34]. The ability to modulate cellular processes under salinity stress requires perception of stress, signal transduction and adaptation to maintain ionic homeostasis [3, 21].

Microorganisms living in the rhizosphere often establish mutualistic relationships with living plants [25]. Rhizobacteria improve plant nutrition and in some cases enhance tolerance to drought and salinity [28, 53]). The role of microorganisms and microorganism-based biostimulants to increase tolerance to abiotic stress has been extensively reviewed [53]. One bacterial species candidate for enhancing abiotic stress tolerance is *Azotobacter chroococcum*. *Azotobacter* is a free-living aerobic rhizobacteria classified as a plant growth-promoting rhizobacteria (PGPR) [54, 55]. Capable of fixing nitrogen, it can stimulate plant growth through nutrient supplementation or through the production of phytohormones such as auxins, gibberellins, and cytokinins (Joseph et al., 2007; [23, 1]). Root growth can be stimulated through rhizobacteria expressing the ACC deaminase, which in turn lowers endogenous ethylene synthesis in roots [45]. Screening of various salt-tolerant strains of *Azotobacter* has revealed that some strains are able to colonize the rhizosphere successfully and promote plant growth in saline soils. One strain in particular, *Azotobacter* strain ST24, was found to enhance growth when applied in conjunction with salt-tolerant wheat varieties [7]. Inoculation of maize plants with *Azotobacter* has been reported to improve growth in control and saline stress conditions [42]. Two salt tolerant strains were also reported to alleviate saline stress by improving sodium exclusion and potassium uptake [42]. Experiments with wheat and inoculation with *A. chroococcum* demonstrated improved phosphorous nutrition, increases in grain yield and root biomass, increased osmotic adjustment and activation ROS response genes [27, 46]. *A. chroococcum* treatments were found to be beneficial to wheat plants under water deficit conditions by increasing total chlorophyll content and relative water content [24]. While most work on the role of *Azotobacter* has been done on cereal crops, evidence for a growth promotion in tomato (F1 Hybrid, GS -15) has only recently been demonstrated [38]. However, the role of *Azotobacter* as stress protectant in tomato has yet to be addressed.

Here we report the findings from a greenhouse experiment using the rhizobacteria *Azotobacter chroococcum* 76A, which was isolated from compost derived from industrial agricultural wastes from olive pomace, industrial sludge from vegetable processing, and borland from the distillation of molasses [40]. The 76A strain has demonstrated tolerance to salt and drought stresses [54]. Using inoculated plants and uninoculated controls, we performed a series of experiments with two levels of salinity stresses (moderate at 50 mM NaCl and severe at 100 mM NaCl) and nutrient regimens, an optimal nutrient regimen and sub-optimal applied at 50% of the optimal concentration and without NH NO_3_). We found that inoculation of Micro Tom tomato plants with the strain *A. chroococcum* 76A facilitated growth and conferred protective effects under both moderate (50 mM NaCl) and severe (100 mM NaCl) salt stress at sub-optimal nutritional levels.

## Methods

### Plant growth conditions

A greenhouse experiment was carried at the experimental station of the University of Naples Federico II, Southern Italy (lat. 43°31’N, long. 14°58′E; alt. 60 m above sea level) with Micro Tom tomato plants. Tomato seeds were germinated in peat on May 2015 and grown until the 3rd-4th true leaf. Plants were transplanted in 10 cm Ø plastic pots at 30 Days-After-Sowing (DAS) containing pure peat moss (100%) and drip irrigated with nutrient solutions from 35 DAS. At transplanting, the growth substrate was inoculated with *Azotobacter chrococcum* 76A strain as described in section “Bacterial strain and inoculum preparation”. The irrigation water was characterized by a high bicarbonate concentration, presence of Na^+^ and Cl^−^ (8.63 mg l^− 1^ Na^+^ and 10.3 mg l^− 1^ Cl^−^) and with values of pH and electrical conductivity (EC) of 7.3 and 0.58 dS m^− 1^, respectively. Plants were fertigated daily using six nutrient compositions, calculated from a standard nutrient solution (SNS) previously used for Micro Tom [29, 44]. The SNS composition was 1.93 mM NO_3_, 2.53 mM P_2_O_5_, 7.64 mM K_2_O, 1.48 mM MgO, 0.84 μM CuEDTA, 10 μM Fe DTPA, 3.45 μM Mn EDTA, 2.08 μM Mo, 0.83 μM Zn EDTA.

The SNS was distributed at two different concentrations, optimal (Opt - 100% SNS augmented with 3.25 mM NH NO_3_) and sub-optimal (Sub - 50% of SNS without NH NO_3_). Opt and Sub solutions were salinized by adding NaCl at concentrations of 50 or 100 mM. Non-salinized controls were provided for both Opt and Sub nutrient solutions. Overall, 6 nutrient solutions were compared: Sub-0NaCl, Sub-50 NaCl, Sub-100 NaCl, Opt-0 NaCl, Opt-50 NaCl, Opt-100 NaCl. The six nutrient solutions were pumped from independent 100 L tanks through a drip-irrigation system, with one emitter per plant (2 l h^− 1^). Two fertilization treatments were applied per day, each of 1–3 min duration. The *A. chroococcum* inoculum [54] was given at 30 and 71 DAS. Non-inoculated plants were included as controls for all treatments.

### Bacterial strain and inoculum preparation

The strain *A. chroococcum* 76A, previously selected for its multiple plant growth promotion activities as well as antimicrobial activity and tolerance to salt and drought stress [2, 54], was used. For inoculum preparation, the strain was grown in Yeast Mannitol (YM) liquid medium at 28 °C for 24 h in a rotary shaker (150 rpm). The culture was harvested at the late exponential phase of growth, centrifuged at 3293 x g, and the bacterial cells were suspended in a 5% sucrose solution at the ratio 1:5 (w:v). The strain was freeze-dried and added to quartz sand to reach a microbial concentration of approximately 1 × 10^7^ CFU g^− 1^. The inoculum was applied in the central part of the pot in the peat substrate before tomato transplanting. For non-inoculated controls, a 5% sucrose solution lacking bacterial cells was applied to substrate similar to inoculated treatment. Viable microbial counts were performed immediately after transplanting and at the end of the experiment (90 DAS). Rhizosphere samples (10 g) were suspended in 90 mL of quarter strength Ringer’s solution (Oxoid, Milan, Italy). After shaking, suitable dilutions (1:10) were performed and used to inoculate nitrogen-free medium LG agar [40] by using the Surface Spread Plate Count Method. The plates were incubated for 48–72 h at 28 °C.

### Biometric and physiological measurements

At the end of the experiment (90 DAS), plants were separated in leaves, stems, roots and fruits for fresh biomass determination and their tissues were dried in a forced-air oven at 80C for 72 h for the dry biomass determination. The final plant height, the number of leaves, fruits, leaf area and number were also recorded. The leaf area was measured with a Li-Cor 3000 area meter (Li-Cor, Lincoln, NE-USA).

After flowering, the following physiological measurements were performed: leaf relative water content (RWC), photosynthetic rate, leaf stomatal conductance, water potential and SPAD index. The RWC was measured on the fully expanded fourth or fifth leaf from the top of the plant. RWC was calculated according to Jones and Turner [22]. The photosynthetic rate (P) and stomatal conductance (gs) were determined with a gas exchange ADS LCA-4 infrared gas analyzer (Analytical Development Company, Hoddesdon, UK). The SPAD index was measured with a SPAD 502-Plus chlorophyll meter (Konica Minolta, New Jersey, USA).

The water potential was measured psychrometrically using a dew-point psychrometer (WP4, Decagon Devices, Pullman, Washington, USA). The osmotic potential (Ψπ) was measured on frozen leaf samples and the pressure potential (Ψp) was estimated as the difference between Ψw and Ψπ, assuming a matric potential equal to zero.

### Mineral analysis

Mineral composition was determined using dried, finely ground (mesh 0.5 mm) samples of leaves and roots. Anions and cations were extracted in Milli-Q water (Merck Millipore, Darmstadt, Germany) in a thermostatic bath at 80 °C for 10 min (ShakeTemp SW22, Julabo, Seelbach, Germany). After centrifuging at 6000 g for 10 min, the supernatant was filtered (0.2 μm) and analyzed by ion chromatography with suppressed conductivity detection using a Dionex ICS-3000 system (Sunnyvale, CA, USA). Cations analysis was carried out with isocratic method (20 mM; flow rate 1 ml/min) using an IonPac CS12A column with a CG12A guard column and methanesulfonic acid as eluent. Anions analysis was performed with NaOH gradient (1 mM – 50 mM; flow rate 1.5 ml/min) using an IonPac AS11HC column with an AG11HC guard column.

### RNA extraction *and qRT-PCR*

Leaves of 12-week-old plants (90 DAS, 19 DAST) from all treatments were harvested and immediately frozen in liquid nitrogen and stored at − 80 °C. Leaves from the same treatment were mixed and three replications per bulk were analyzed. 100 mg of fresh leaf tissue per sample was homogenized with liquid nitrogen and extracted with 1 ml of TRIzol (Life Technologies, Carlsbad, CA, USA). First-strand synthesis was performed with a QuantiTect Reverse Transcription Kit (QIAGEN, Valencia, CA, USA) using 1 μg of RNA. Real-time qPCR reactions, using 10 ng of cDNA per reaction, two experiments, four replicates per experiment, were carried out on an ABI Instruments 7900HT qPCR detection system (Applied Biosystems, Foster City, CA, USA) using Platinum SYBR Green qPCR SuperMix-UDG with ROX (Life Technologies, Carlsbad, CA, USA). All qRT-PCR primers were determined to be within 3% efficiency of each other. Relative expression levels were calculated using *Actin2* as an internal standard and the ΔΔCt method for relative quantification. The primers used in this study are listed in Additional file 1: Tables S1.

### Statistical analysis

Data were analyzed with a two way ANOVA (Nutrient solution x Inoculum). Least Significant Difference (LSD) multiple range comparison tests were used to determine differences between means (*P* ≤ 0.05). For gene expression analysis, the ΔCt values of each gene of interest and the actin reference gene were analyzed using Student’s T-test. Single asterisks denote significant differences according to Student (*P* < 0.1) between untreated controls and inoculated, double asterisks denote (*P* < 0.05) and triple asterisks denote (*P* < 0.01) between untreated controls and inoculated plants.

## Results

### Bacterial growth

The strain *A. chroococcum* 76A, inoculated at a concentration of approximately 6 Log CFU g^− 1^, was able to grow under all experimental conditions. The diazotrophic strain growth significantly increased at the end of the experiment reaching values of approximately 7 Log CFU g^− 1^ (Table 1). Specifically, the highest microbial concentration (from 7.80±0.05 to 7.94 ±0.08 Log CFU g^− 1^) was recovered in the rhizosphere of tomato plants cultivated under sub-optimal nutrient solution (50% standard nutrient solution) regardless of the salt stress applied (Table 1). This result implied that salt stress (0, 50 and 100 mM NaCl) did not exert negative effects on microbial growth. Interestingly, it was observed that conditions with optimal levels of nitrogen significantly limited microbial growth (Table 1).

**Table 1:** Enumeration of autochthonous free-living N_2_-fixing bacteria and *Azotobacter chroococcum* 76A in the rhizosphere of non-inoculated (C) and inoculated (I) tomato plants at the end of cultivation cycle

Treatment	Free-living N_2_-fixing bacteria (Log CFU/g)	*A. chroococcum* 76A (Log CFU/g)
(C) Optimal N, 100 mM NaCl	6.83 ± 0.13^c^	–
(C) Optimal N, 50 mM NaCl	6.63 ± 0.30^c^	–
(C) Optimal N, 0 mM NaCl	6.77 ± 0.07^c^	–
(C) Sub-Optimal N, 100 mM NaCl	6.80 ± 0.18^c^	–
(C) Sub-Optimal N, 50 mM NaCl	6.66 ± 0.31^c^	–
(C) Sub-Optimal N, 0 mM NaCl	6.74 ± 0.24^c^	–
(I) Optimal N, 100 mM NaCl	6.72 ± 0.20^c^	7.67 ± 0.04^b^
(I) Optimal N, 50 mM NaCl	6.67 ± 0.18^c^	7.58 ± 0.06^b^
(I) Optimal N, 0 mM NaCl	6.81 ± 0.13^c^	7.59 ± 0.09^b^
(I) Sub-Optimal N, 100 mM NaCl	6.50 ± 0.16^c^	7.81 ± 0.05^a^
(I) Sub-Optimal N, 50 mM NaCl	6.58 ± 0.07^c^	7.80 ± 0.05^a^
(I) Sub-Optimal N, 0 mM NaCl	6.51 ± 0.15^c^	7.94 ± 0.08^a^

Different letters after the values indicate significant differences (Tukey’s HDS post hoc test, *P* < 0.05)

The values represent the means ± SD of three replicates of three independent experiments

### Plant growth responses and fruit yield

**Fig. 1:**
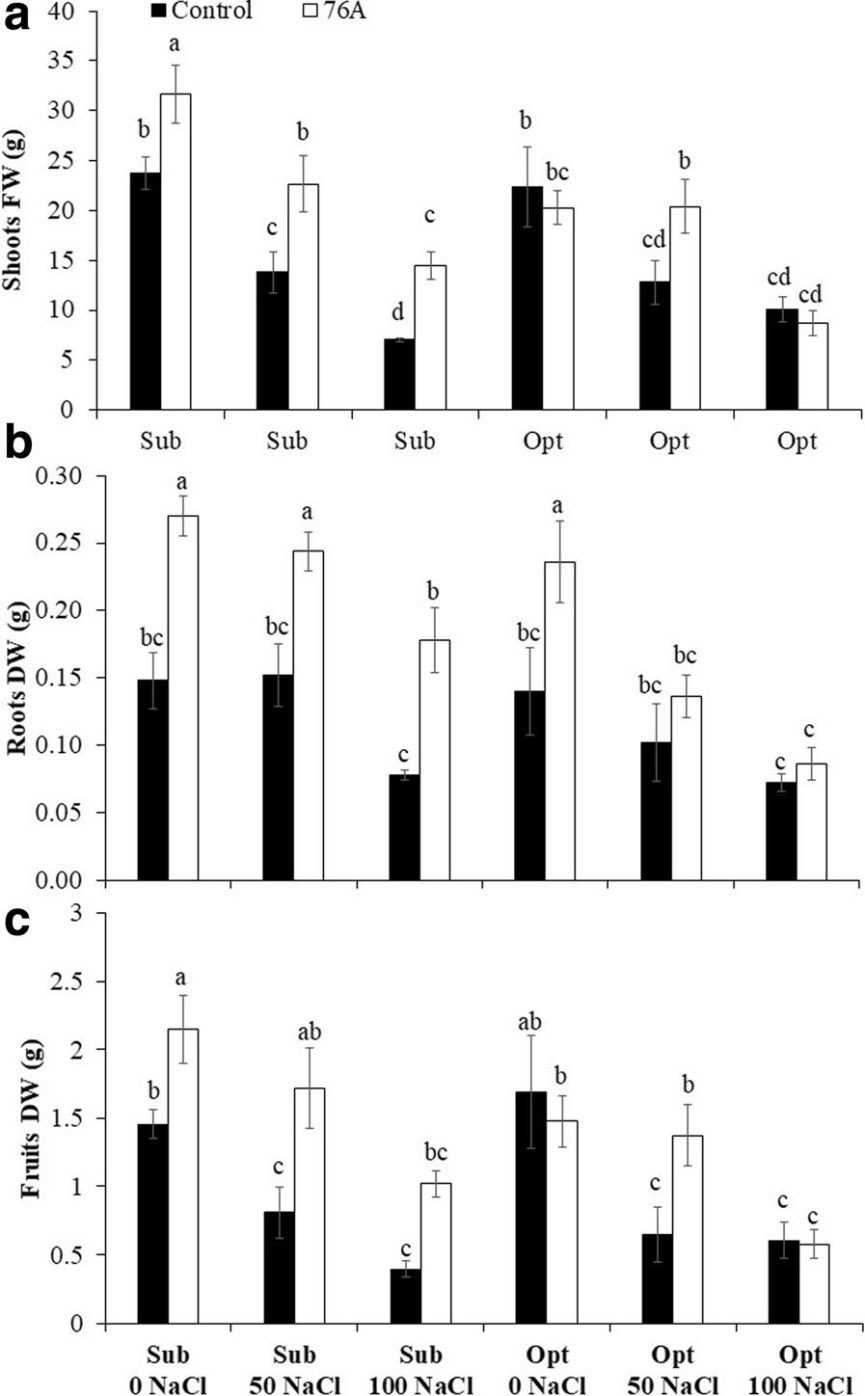
Effect of *Azotobacter chroococcum* 76A on biometric parameters of Micro Tom grown under increasing salinity (0, 50, 100 mM NaCl) and two nutrient regimens (Sub – *Suboptimal*, Opt- *Optimal*). **a** Shoot Fresh Weight, (**b**) Roots dry weight, (**c**) Fruits dry weight. Vertical bars indicate ± SE of means, different letters denote significant differences between uninoculated controls and plants inoculated with *A. chroococcum* 76A according to Least Significant Different (LSD) multiple range comparison test

Inoculation (I) with *A. chroococcum* 76A and nutrient solutions (N) had both a remarkable effect on plant growth and fruit yield, with a significant interaction (IxN) for some of the biometric parameters considered (Table 2). Shoot dry weight (SDW), fruit fresh weight (FFW) and fruit number per plant (FN) all increased, compared to untreated control plants, upon microbial inoculation (+ 45% SDW, + 39% FFW, + 49% FN). These parameters were also similarly affected by different nutrient solutions (Table 2). The addition of NaCl caused a decline in terms of plant growth and yield regardless of the nutritional level. At 50 mM and 100 mM NaCl, the average SDW, FFW and FN reduction vs. non-salinized solutions was − 15% and − 44% (for SDW), − 37% and 64% (for FFW), − 31% and − 53% (for FN), respectively (means of Sub + Opt nutritional regimens). It should be noted that in the absence of NaCl, the fruit number (9.3 vs. 8.3) at sub-optimal vs. optimal nutritional levels was similar, whereas SDW and FFW were reduced by 21% and 25% respectively in optimal conditions. These results indicate that higher nutrient concentration and additional nitrogen provided as NH NO with the optimal nutritional regimen did not further improve the number of fruit per plant and it was indeed inhibitory for shoot and fruit growth. A significant interaction between microbial inoculation (I) and nutrient solutions (N) was found for shoot fresh weight (SFW), root dry weight (RDW) and Fruit Dry Weight (FDW) (Fig. 1). For these parameters, although it was observed an overall proportional decline with salinity under both sub-optimal and optimal nutritional regimen, there were substantial differences between inoculated plants and non-inoculated controls. Specifically, at moderate (50 mM NaCl) and severe (100 mM NaCl) salinity, *A. chroococcum* 76A treated plants performed always better than uninoculated plants under sub-optimal nutritional regimen in terms of SFW (+ 44% as average of the two salinity treatments), RDW (+ 45%) and FDW (+ 56%). Higher nutrients concentration and addition of NH NO (optimal regimen) did not further improve the overall plant performance and it indeed flattened down the differences between *A. chroococcum* 76A and uninoculated plants. The optimal nutritional level and most likely the extra nitrogen caused a partial inhibition of *Azotobacter* growth (Table 1) and, consequently, its effects on some growth parameters (Fig. 1).

**Table 2:** Effect of *Azotobacter chroococcum* 76A on the main biometric parameters of Micro Tom grown under increasing salinity (0, 50, 100 mM NaCl) and two nutrient regimens (Sub – *Suboptimal*, Opt- *Optimal*)

	Shoots FW	Shoots DW	Roots DW	Fruits FW	Fruits number	Fruits DW	Leaves FW
	g	g	g	g		g	g
Inoculum (I)							
Control	15.0 b	1.1 b	0.1 b	9.6 b	5.1 b	0.9 b	0.7 b
76 A	19.7 a	1.6 a	0.2 a	13.3 a	7.6 a	1.4 a	1.0 a
Nutrent Solution (N)							
Sub - 0 mM NaCl	27.7 a	1.9 a	0.2 a	19.8 a	9.3 a	1.8 a	1.1 a
Sub - 50 mM NaCl	18.2 b	1.7 ab	0.2 a	11.3 bc	6.4 b	1.3 b	0.9 ab
Sub - 100 mM NaCl	10.7 c	1.0 c	0.1 b	6.3 d	4.2 c	0.7 c	0.6 b
Opt - 0 mM NaCl	21.4 ab	1.5 b	0.2 a	14.8 b	8.3 a	1.6 ab	1.0 a
Opt - 50 mM NaCl	16.6 b	1.2 bc	0.1 b	10.5 c	5.8 b	1.0 b	0.7 b
Opt - 100 mM NaCl	9.4 c	0.9 c	0.1 c	6.0 d	4.0 c	0.6 c	0.5 b
Significance							
I	***	***	***	***	***	***	*
N	***	***	***	***	***	***	***
IxN	*	ns	*	ns	ns	*	ns

ns, *; **, ***Non significant or significant at *P* ≤ 0.05, 0.01, and 0.001, respectively

### Gas exchange analysis, water relations and SPAD

With respect to leaf water potential and gas exchanges (Table 4), there were no differences between control and *A. chroococcum* 76A treated plants, whereas salinity reduced photosynthesis under sub-optimal nutritional regimen (− 50% at 100 mM NaCl). The photosynthetic levels under optimal nutritional regimen were generally lower than those at suboptimal nutrition and they did not differ at varying salinity. RWC and SPAD values were 5% and 15% higher in *A. chroococcum* 76A treated than uninoculated control plants, respectively. These parameters also decreased with salinity under both suboptimal and optimal nutritional levels. In the absence of NaCl, the RWC under sub-optimal nutritional level was 7% higher than under optimal nutritional regimen (compare Sub-Opt 0 mM NaCl vs. Opt 0 mM NaCl in Table 4).

### Leaf ion contents

**Fig. 2:**
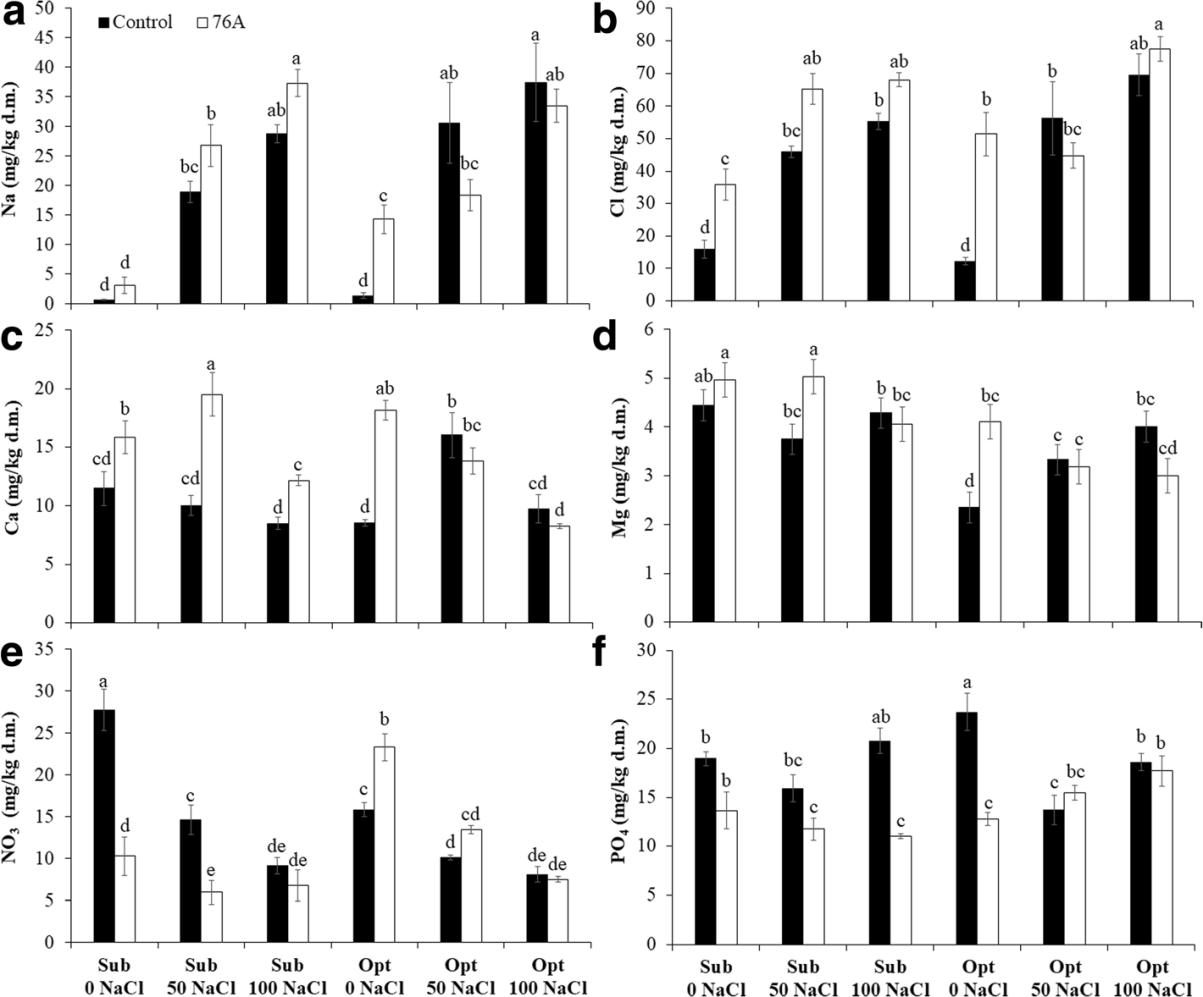
Effect of *Azotobacter chroococcum* 76A on the leaves ions content of Micro Tom grown under increasing salinity (0, 50, 100 mM NaCl) and two nutrient regimens (Sub – *Suboptimal*, Opt- *Optimal*). **a** Na^+^, (**b**) Mg ^2+^, (**c**) Ca^2+^, (**d**) Cl^−^, (**e**) NO_3_^−^, (**f**) PO_4_^−^. Vertical bars indicate ± SE of means, different letters denote significant differences between uninoculated controls and plants inoculated with *A. chroococcum* 76A according to Least Significant Different (LSD) multiple range comparison test

The leaf ion profile was significantly altered in response to both microbial inoculation (I) and nutritional regimen (N) (Table 3). Leaf ammonia concentration was higher under optimal fertilization (3.1 mg/kg dry matter) compared to the sub-optimal regimen (0.8 mg/kg dry matter). Potassium decreased with respect to both the microbial treatment (− 15%) and nutritional regimens with a 36% and 45% lower K tissue concentrations at 50 and 100 mM NaCl, respectively, compared to non-salinized solutions as average of sub-optimal and optimal nutritional regimen, (Table 3). For all other ions, a significant interaction was found between microbial treatment and nutritional regimen (Fig. 2). Na^+^ and Cl^−^ levels in leaves increased upon addition of NaCl to the nutrient solution under both sub-optimal and optimal nutritional regimens. However, the concentrations of these ions were remarkably higher in *A. chroococcum* 76A treated plants vs. uninoculated controls in the absence of NaCl stress with + 91% for Na^+^, under optimal nutrient regimen (Fig. 2a) and + 56% and + 76% for Cl^−^ under sub-optimal and optimal nutritional regimens, respectively (Fig. 2b). Ca^2+^ was always higher in *A. chroococcum* 76A plants under sub-optimal nutritional levels (Fig. 2c). The differences between inoculated plants and controls were generally attenuated under optimal nutritional regimen, although in the absence of NaCl the leaf Ca^2+^ level of inoculated plants was still twice that of uninoculated controls. Mg^2+^ levels were rather stable in non-inoculated plants under all nutritional regimens, with a moderate decline under optimal nutritional conditions, in the absence of NaCl. 76A treated plants again performed slightly better under sub-optimal nutritional regimen with highest values at 0 and 50 mM NaCl compared to all other treatments (Fig. 2d). NO_3_^−^ levels generally declined with salinization and were lower in 76A plants vs. uninoculated controls at 0 and 50 NaCl (− 63% and − 59%, respectively) (Fig. 2e). This pattern was somehow reverted under optimal fertilization, at least in the absence of NaCl (+ 32% in 76A plants). For PO_3_ ions, we found similar levels in inoculated vs. non-inoculated plants at all nutrient solutions tested, with the exception of significant 47% and 46% lower levels at sub-optimal nutritional level + 100 mM NaCl and at optimal nutritional level in the absence of salt (Fig. 2f).

**Table 3:** Effect of *Azotobacter chroococcum* 76A on the leaves ions of Micro Tom grown under increasing salinity (0, 50, 100 mM NaCl) and two nutrient regimens (Sub – *Suboptimal*, Opt- *Optimal*)

	Na	NH_4_	K	Mg	Ca	Cl	NO_3_	PO_4_
	mg/kg d.m.	mg/kg d.m.	mg/kg d.m.	mg/kg d.m.	mg/kg d.m.	mg/kg d.m.	mg/kg d.m.	mg/kg d.m.
Inoculum (I)								
Control	19.6	2.6	29.1 a	3.7 b	10.7 b	42.5 b	14.3 a	18.6 a
76 A	22.2	2.3	24.7 b	4.1 a	14.6 a	57.1 a	11.2 b	13.7 b
Nutrient Solution (N)								
Sub - 0 mM NaCl	1.8 c	0.8 c	42.8 a	4.7 a	13.7 a	25.9 d	19.0 a	16.3 ab
Sub - 50 mM NaCl	22.8 b	1.2 c	23.9 c	4.4 a	14.8 a	55.6 bc	10.3 bc	13.8 c
Sub - 100 mM NaCl	33.0 a	1.4 c	21.2 cd	4.2 a	10.3 b	61.6 b	7.9 c	15.9 b
Opt - 0 mM NaCl	7.8 c	3.1 b	30.9 b	3.2 b	13.4 a	31.8 d	19.6 a	18.3 a
Opt - 50 mM NaCl	24.5 b	3.0 b	23.6 c	3.3 b	14.9 a	50.5 c	11.8 b	14.6 bc
Opt - 100 mM NaCl	35.5 a	5.2 a	19.1 d	3.5 b	9.0 b	73.5 a	7.8 c	18.2 a
Significance								
I	ns	ns	**	***	***	***	***	***
N	***	***	***	***	**	***	***	**
IxN	*	ns	ns	***	**	***	***	***

ns, *; **, ***Non significant or significant at *P* ≤ 0.05, 0.01, and 0.001, respectively

### Gene expression

Four genes were chosen to correlate the rates of nitrogen content to molecular mechanisms that play a role in nitrogen uptake: the NH_4_^+^ transporter (*AMT2*), the NO_3_ transporter (*NRT2.1*), nitrite reductase (*Nii*), and nitrate reductase (*NR*) The plasma membrane sodium antiporter, *SALT OVERLY SENSITIVE 1* gene (*SOS1*), two members of the Na^+^-K^+^/H^+^ antiporter family (*NHX1* and *NHX2*), and the Na^+^-selective class I-HKT transporter (*HKT1;1*) were used to assess ion transport. Optimal N levels led to a general decreased expression of *AMT2, NRT2.1* and *NR* in leaves (Fig. 4). For these genes, inoculation with *A. chroococcum* 76A did not induce notable changes to expression under sub-optimal N, with the exception of *NRT2.1* whose relative expression was less than half of the untreated control at moderate and severe salinity (Fig. 4). *The LATE EMBRYOGENESIS ABUNDANT* (*LEA*) gene was chosen for its high inducibility under both salt and drought stress as previously reported [19]. *LEA* expression in inoculated plants under stress treatment was higher than in uninoculated, implying a level of crosstalk between signaling and nutrition. *LEA* serves as an indicator of stress response and the increased expression levels seen at optimal N conditions indicate that additional nitrogen exacerbated the stress phenotype. The expression of the plasma membrane sodium antiporter, *SOS1* was also evaluated. *SOS1* is the primary transporter responsible for exclusion of sodium ions from the cytoplasm and required for salt tolerance [17]. Expression in sub-optimal conditions was slightly lower in inoculated plants (Fig. 4). Conversely, in the optimal nutrient regimen, expression of *SOS1* was higher in inoculated plants. A second sodium transporter, the Na^+^-selective class I-HKT transporter (*HKT1;1*), was selected to evaluate the ion homeostasis (Ansins, et al., 2012). *HKT1;1* expression was not induced upon inoculation with *A. chroococcum* 76A or by salt stress in sub-optimal nutrient condition in leaves of MicroTom plants. Expression of *HKT1;1* was slightly increased in optimal nutrients and upon *Azotobacter* inoculation (Additional file 1: Figure S1). Finally, expression of *NHX1* and *NHX2* showed increases in all salt stress conditions, with no remarkable differences between inoculated and uninoculated plants (Additional file 1: Figure S1).

## Discussion

### *A. chroococcum* 76A is an effective salt stress protectant

Optimizing fertilization techniques to reduce the environmental impact of chemical fertilizers is critical for developing sustainable agricultural systems ([13]; Dadkhah, 2013). *Azotobacter* based biofertilizers, alone or in combination with other microbial-based products, show promise as alternatives to chemical fertilization, especially in organic production [51]. Data on the effects of rhizobacteria applications on tomato performance are limited [33, 38]. Here we provide evidence for remarkable effects of *A. chroococcum* 76A on plant growth and yield at reduced nutritional level (Table 2 and Fig. 1). The strain *A. chroococcum* 76A has been previously characterized to be tolerant to desiccation and as halotolerant, capable of growing in high salinity medium, at concentrations which would be lethal for other bacterial strains [54]. Furthermore, the 76A strain was shown to form association with tomato roots in vitro. This strain exerted multiple plant growth promotion activities including indole-3-acetic acid and siderophore production, phosphate solubilization, and ACC deaminase activity [54]. Based on these properties, we hypothesized that root inoculation with the halotolerant 76A strain may confer benefits in terms of nitrogen availability and salt tolerance in tomato. The presence of moderate and severe salt stress did not inhibit the growth of the 76A strain in the soil while higher N content had a slight inhibitory effect on growth (Table 1), as previously reported for *Azotobacter* ([5, 36]). In this study we observed that in tomato, *A. chroococcum* 76A acted as a general growth enhancer (Table 2). Similar results in terms of plant growth and yield have been reported by El-Shanshoury et al. [14] for dry weights of shoots and roots, + 84% and + 200% respectively in inoculated plants, and in terms of fruit quality [38]. Use of *Azotobacter* strains in field trials has also indicated that inoculation increases growth, particularly when modest nitrogen supplementation was used [47]. In contrast, our results demonstrate that plants grown at sub-optimal nutritional conditions and in the presence of *A. chroococcum* 76A performed better than any other treatment in term of shoot fresh weight, root dry weight and fruit dry weight (Fig. 1). This indicates the outstanding potential of this strain as potential substitute of chemical fertilization, beyond its current role as a complementary additive. This function seemed to be partially repressed at optimal nutritional levels (Fig. 1), and is likely linked to the inhibitory effect of higher N concentrations on bacterial growth (Table 1). We cannot rule out, however, the possibility that the optimal nutritional regimen used in our study may have resulted in some toxicity level to plants in our experimental conditions. Remarkably, inoculation with the strain *A. chroococcum* 76A in our experiments consistently increased yield in terms of fruit fresh weight and number. In contrast, under optimal nutritional conditions, inoculation improved fruits dry weight only at moderate salt stress (Fig. 1). These results further indicate that *A. chroococcum* 76A, in terms of growth and yield promotion, is more suited to sub-optimal nutritional conditions and increased levels of nitrogen present in the optimal nutritional treatment were inhibitory in terms of yield (Fig. 1). The effects of *A. chroococcum* on plant growth have been associated with the production of auxins, cytokinins, and GA like molecules which all have well established functions in plant growth regulation [55]. In our experimental conditions gas exchange analysis did not reveal differences in *A. chroococcum* 76A treated vs. control plants. It must be noted that gas exchanges and water relation parameters were generally low at time of measurement (after flowering) when we observed incipient leaf senescence. Nevertheless, inoculated plants had higher Relative Water Content (RWC) suggesting that the microbial activity may have triggered the production and/or uptake of osmolytes that may have contributed to maintain a favorable water uptake (Table 4) with beneficial effects on cellular turgor and photosynthetic system. A further, yet indirect, indication of the protective effects of *A. chroococcum* 76A on the photosynthetic machinery was provided by the SPAD results (Table 4). The Single Photoelectric Analyzing Diode (SPAD) meter is used for fast, reliable and non-destructive measurement of leaf chlorophyll levels [31]. All SPAD values measured for unstressed plants were in the range of 40–50, close to other reported values for tomatoes grown under similar conditions [18, 26]. We observed that *A. chroococcum* 76A treated plants demonstrated higher values than uninoculated controls, suggesting a protective effect of *A. chroococcum* 76A treatment on the photosynthetic machinery.

**Table 4:** Effect of *Azotobacter chroococcum* 76A on the Relative Water Content (RWC), SPAD index, Water Potential, Net Photosynthesis (P), Stomatal Conductence (gs) of Micro Tom grown under increasing salinity (0, 50, 100 mM NaCl) and two nutrient regimens (Sub – *Suboptimal*, Opt- *Optimal*)

	P	gs	Water Potential	RWC	SPAD
	μmol m^−2^ s^−1^	mol m^−2^ s^−1^	MPa	%	
Inoculum (I)					
Control	1.5	0.013	−2.3	67.7 b	45.4 b
76 A	1.2	0.017	−2.2	71.0 a	52.3 a
Nutrent Solution (N)					
Sub - 0 mM NaCl	2.0 a	0.020	−2.1	76.8 a	51.8 ab
Sub - 50 mM NaCl	1.8 a	0.015	−1.9	66.2 c	50.7 b
Sub - 100 mM NaCl	1.0 b	0.013	−1.8	68.7 bc	45.6 c
Opt - 0 mM NaCl	1.1 b	0.018	−2.5	71.3 b	54.3 a
Opt - 50 mM NaCl	0.8 b	0.010	−2.6	66.1 c	50.2 b
Opt - 100 mM NaCl	1.1 b	0.020	−2.7	67.1 c	40.7 c
Significance					
I	ns	ns	ns	**	***
N	**	ns	ns	***	***
IxN	ns	ns	ns	ns	ns

ns, *; **, ***Non significant or significant at *P* ≤ 0.05, 0.01, and 0.001, respectively

### Ionic profile reveals a stress pre-adaptation state of *A. chroococcum* 76A treated plants

**Fig. 3:**
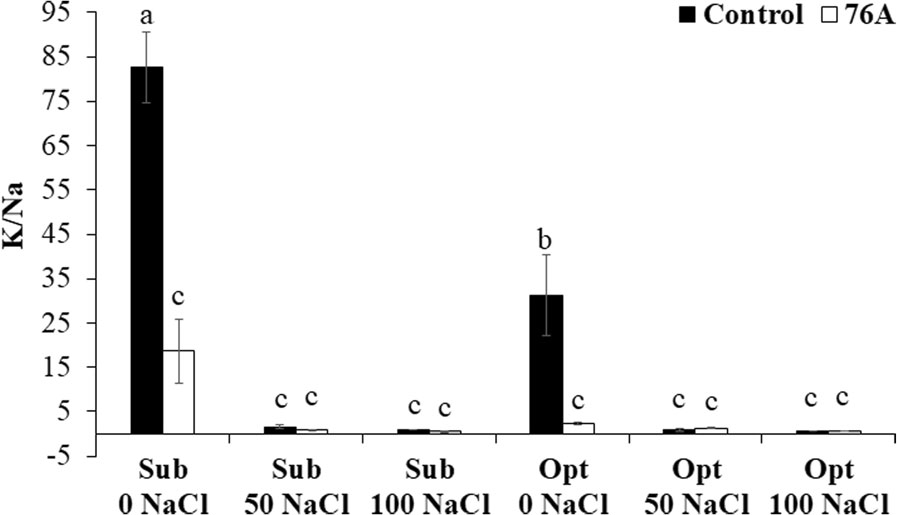
Effect of *Azotobacter chroococcum* 76A on the leaves K/Na ratio of Micro Tom grown under increasing salinity (0, 50, 100 mM NaCl) and two nutrient regimens (Sub – *Suboptimal*, Opt- *Optimal*)

**Fig. 4:**
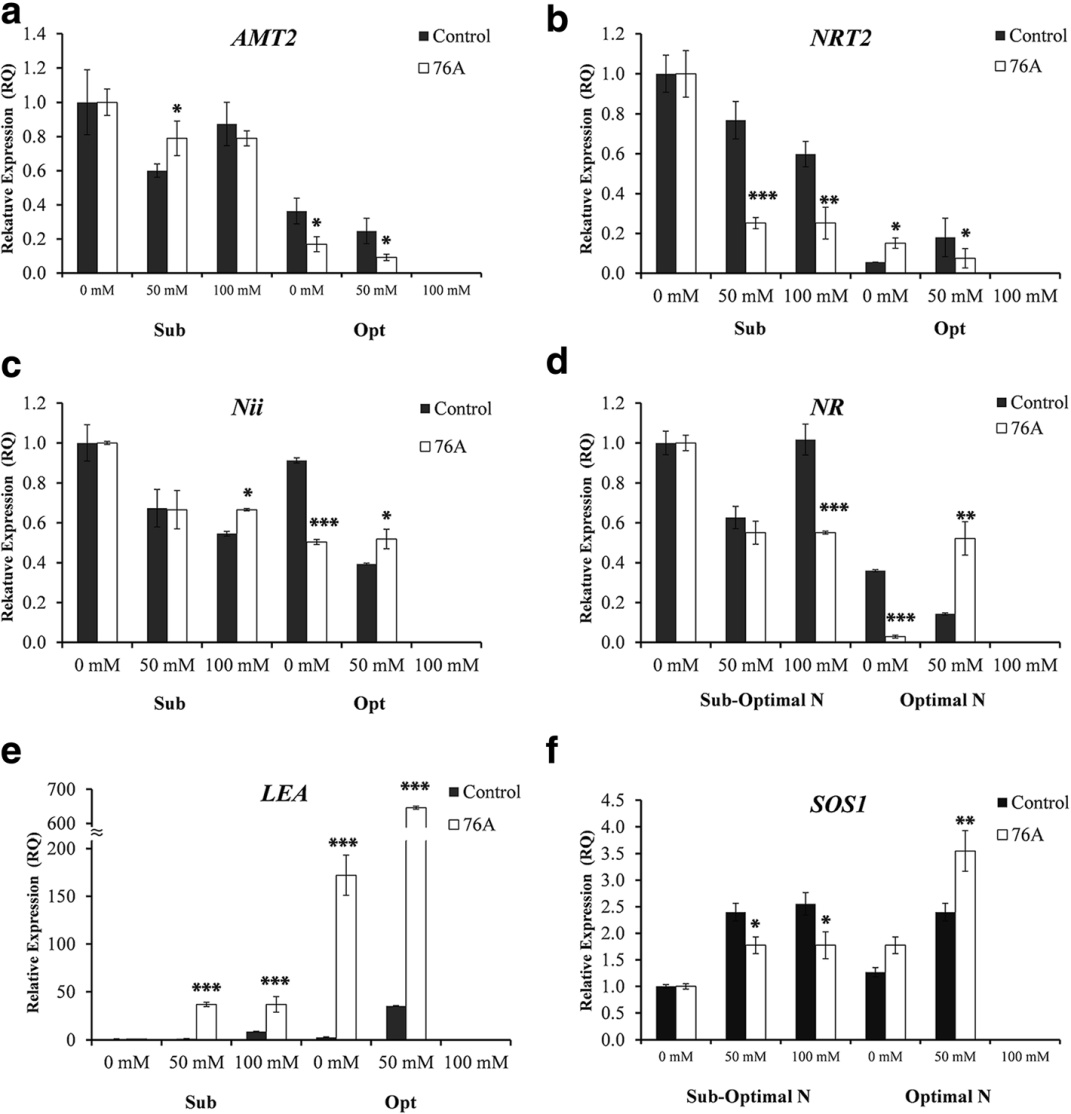
Gene expression of key nitrogen assimilation enzymes and abiotic stress marker. **a** AMT2, (**b**) NRT2, (**c**) Nii, (**d**) NR, (**e**) LEA, (**f**) SOS1 relative Expression (RQ) quantified by qRT-PCR in samples of uninoculated (control) and inoculated (76A) plants treated with: 0 mM, 50 mM, and 100 mM NaCl. Single asterisks denote significant differences according to Student (*P* < 0.1) between untreated controls and inoculated, double asterisks denote (*P* < 0.05) and triple asterisks denote (*P* < 0.01) between untreated controls and inoculated plants

Tomato plants treated with *A. chroococcum* 76A had higher levels of Na^+^ and Cl^−^ ions compared to their relative untreated controls (Table 3). The bacterial inoculum seemed to have a tissue concentration effect on the low levels of Na^+^ and Cl^−^ ions dissolved in non-salinized irrigation water (Table 3; Fig. 2a, b). This increase mirrored the increase of Ca^2+^ (Fig. 2c) that typically is associated with protection from osmotic and ionic stress [56]. While Na^+^ ions may serve as cheap osmoticum in stress adaptation [30] and may have stimulated root growth (Fig. 1b), Ca^2+^ is a fundamental component of the regulatory machinery for cellular ion homeostasis during salinity stress but also hormonal regulation and growth control [4]. The increase of cytosolic Ca^2+^ (which was 30% higher in 76A plants vs. control plants) under salt stress triggers the SOS pathway, through the activation of Ca^2+^-binding protein SOS3 and the kinase SOS2, which, upon SOS3 activation, form a complex that stimulates activity of the Na^+^/H^+^ exchanger SOS1. Activation of SOS1 in turn will remove Na^+^ from the cytoplasm relieving its toxic effects [8]. High apoplastic Na^+^ would serve as osmoticum without impairing cellular functions [17]. Consistent with this mechanism of Na^+^ detoxification, expression of *SOS1* increased in all salt stress treatments. However, under sub-optimal nutrient regimen, plants inoculated with 76A had lower levels of *SOS1* expression than uninoculated controls (Fig. 4) and accumulated more sodium in salt stress conditions. Lower levels of *SOS1* expression have been shown to increase root and shoot accumulation of sodium in tomato [37]. While salt stress reduced the K^+^/Na^+^ ratio under both nutritional regimens (Fig. 3), the only notable differences in this ratio between controls and inoculated plants were observed in non-stress conditions. In the absence of stress, plants inoculated with *A. chroococcum* 76A had much lower K^+^/Na^+^ ratios than controls. The strain *A. chroococcum* 76A appears to affect the K^+^/Na^+^ ratio by increasing the sodium content. However, this effect was not observed when salt stress was imposed. The mechanism responsible for increased Na^+^ and Cl^−^ is unclear, but it does not appear to inhibit growth in control conditions and interestingly does not come into play under moderate or severe salt stress. A side effect of high leaf Cl^−^ was a reduced PO_4_^−^concentration (Fig. 2f), possibly due to competition between these two anions [9, 41]. Consistent with the PO_4_^−^ pattern, NO_3_^−^ also decreased in sub-optimal nutritional conditions (Figs. 2e). Low NO_3_^−^ levels have been associated to high Na^+^ and Cl^−^ availability/uptake in/from the root zone [10, 49]. However nitrate increased in response to optimal N with inoculated plants containing more nitrate than controls in unstressed conditions. *A. chroococcum* 76A may have likely facilitated assimilation of nitrogen although high salinity was observed to inhibit increased nitrate content. Synergistic effects when nitrogen supplementation is used in combination with Azotobacter strains have been reported [47].

### Molecular mechanisms underlying ion partitioning and *A. chroococcum* 76A induced stress protection

*AMT2* expression was barely detectable under optimal nutritional regimen and moderate salt stress (Fig. 4). Nitrogen transporters such as, *NRT2.1* and *AMT2* as well as *Nii* and *NR* are known to be transcriptionally down regulated by high levels of N and metabolites such as amino acids [32]. In sub-optimal nutritional conditions, inoculation did not significantly increase plant NH_4_^+^ content. Under carbon limiting conditions, uptake of both amino acids and ammonium may be down regulated. The primary uptake of nitrogen into the root cells may be in the form of amino acids instead of NO_3_^−^ and NH_4_^+^ [6]. The presence of *A. chroococcum* 76A appears to synergistically enhance the expression of *LEA* under salt stress, with higher expression levels observed in inoculated plants. Interestingly, under optimal nutritional conditions, expression was elevated even in unstressed plants, with drastically higher levels of expression under salt stress conditions. Possibly the optimal nutrient solution we used, which was adapted from standard solution for soil-less production of cherry tomato plants which are larger and typically have an indeterminate growth, was not ideal for the smaller determinate MicroTom tomato in our specific experimental conditions.

The presence of *A. chroococcum* 76A appears to prime plant responses to stress. Instances of this PGPR increasing stress tolerance have been documented, but the molecular mechanisms remain unknown [48, 50, 58]. Stress priming for salt tolerance has been observed in wheat seed treated with Nitragin biofertilizer, a mixture of *Azotobacter* spp., *Azospirillum* spp. and *Pseudomonas* spp. Inoculation increased also germination under saline conditions [15]. Our results indicate that inoculation increases expression of at least one key gene (*LEA*) involved in salt and drought responses. Inoculation with 76A did not appear to affect the expression of the two evaluated NHX genes (Additional file 1: Figure S1) and we observed no significant differences in K^+^ accumulation in inoculated plants. Sodium accumulation (Fig. 2) corresponded with the expression patterns of the sodium extruder, *SOS1*, with lower levels of expression in sub-optimal nutrients in inoculated plants and higher levels of expression in optimal nutrients. The sodium transporter *HKT1;1* was not significantly affected by inoculation but has been previously reported to not be highly inducible in leaves of tomato plants [52]. Inoculation with 76A appears to alter sodium transport while not affecting potassium uptake. Overall, ion compartmentation could be one mechanism that has contributed in 76A plants to better perform under salt stress (see moderately higher Na^+^ and Cl^−^ associated with better growth), whereas tissue Ca^2+^ accumulation may have played a predominant signaling role in growth control.

## Conclusions

Throughout the literature, the ability of PGPRs to enhance growth is clearly demonstrated in a number of species. It is also becoming clear that PGPRs also have the ability to enhance tolerance to both abiotic and biotic stress. In this study, we show that the *A. chroococcum* 76A strain enhances tolerance to salinity in Micro Tom tomato. We also observed stress priming in plants inoculated with *A. chroococcum* 76A increasing expression of key stress-related genes (*LEA*). The application of optimal nutritional levels appears to be inhibitory to the growth promoting and stress protective effects of *A. chroococcum* 76A. Use of this PGPR may be ideal for low-input agricultural systems where large quantities of chemical fertilizer may not be readily available, portable, or affordable. A small bag of inoculum may prove far more accessible and affordable to a small-scale grower in a developing nation than nitrogen fertilizer derived from the Haber–Bosch process which requires fossil fuels. In the growing context of developing sustainable systems, using PGPRs may provide a means of increasing available nutrients to crop systems as well as increasing tolerance to abiotic stress.

## Additional file

Additional file 1: **Figure S1.** Gene Expression of ion transporters. (A) NHX1, (B) NHX2, (C) HKT1;1, relative Expression (RQ) quantified by qRT-PCR in samples of uninoculated (control) and inoculated (76A) plants treated with: 0 mM, 50 mM, and 100 mM NaCl. Single asterisks denote significant differences according to Student (*P* < 0.1) between untreated controls and inoculated, double asterisks denote (*P* < 0.05) and triple asterisks denote (*P* < 0.01) between untreated controls and inoculated plants. **Table S1.** Primers used in this study. (DOCX 257 kb)

## Abbreviations

76A: *A. chroococcum* 76A
ACC: 1-Aminocyclopropane-1-Carboxylate
AMT: NH_4_^+^ transporter
CFU: Colony Forming Unit
DAS: Days After Sowing
DAST: Days After Stress Treatment
EC: Electrical conductivity
FDW: Fruit Dry Weight
FFW: Fruit Fresh Weight
FN: Fruit Number
I: Inoculated
LEA: Late Embryogenesis Abundant
N: Nutrition
Nii: Nitrite Reductase
NR: Nitrate Reductase
NRT: NO_3_ transporter
Opt: Optimal Nitrogen Nutrient Solution
RDW: Root Dry Weight
ROS: Reactive Oxygen Species
RWC: Relative Water Content
SDW: Shoot Dry Weight
SFW: Shoot Fresh Weight
SOS: Salt Overly Sensitive
SPAD: Single Photoelectric Analyzing Diode
Sub: Sub-Optimal Nitrogen Nutrient Solution
YM: Yeast Mannitol
