# Supplementary material for: Root inoculation with Azotobacter chroococcum 76A enhances tomato plants adaptation to salt stress under low N conditions
Source: BMC Plant Biol. 2018 Sep 20;18:205. doi: 10.1186/s12870-018-1411-5 (PMC6149061; doi:10.1186/s12870-018-1411-5)
Supplement: Supplementary file 1 — Figure S1. Gene Expression of ion transporters. (A) NHX1, (B) NHX2, (C) HKT1;1, relative Expression (RQ) quantified by qRT-PCR in samples of uninoculated (control) and inoculated (76A) plants treated with: 0 mM, 50 mM, and 100 mM NaCl. Single asterisks denote significant differences according to Student (P < 0.1) between untreated controls and inoculated, double asterisks denote (P < 0.05) and triple asterisks denote (P < 0.01) between untreated controls and inoculated plants. Table S1. Primers used in this study. (DOCX 257 kb) [file 12870_2018_1411_MOESM1_ESM.docx]

*

**A**

**B**

**C**


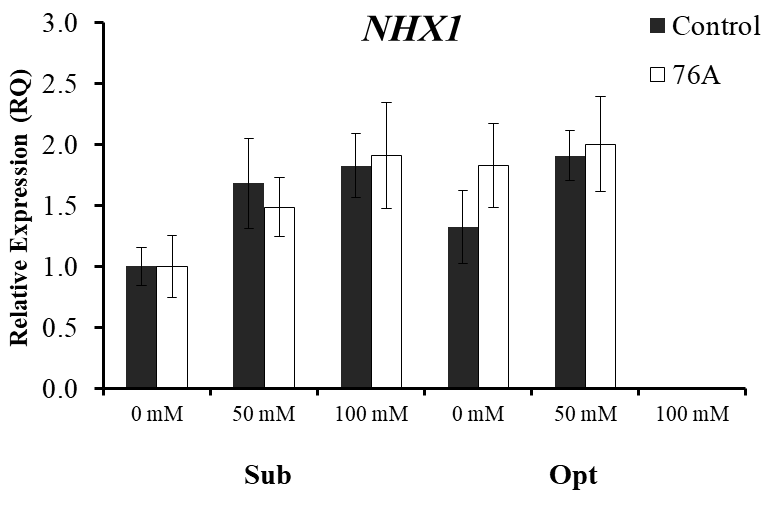

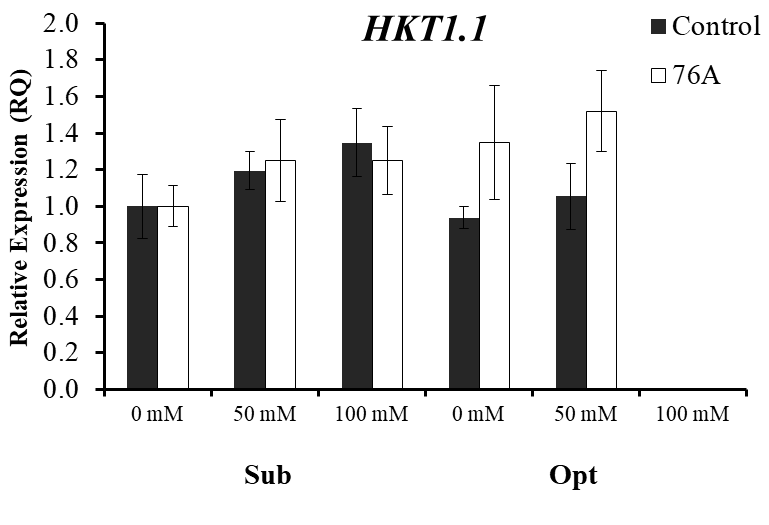

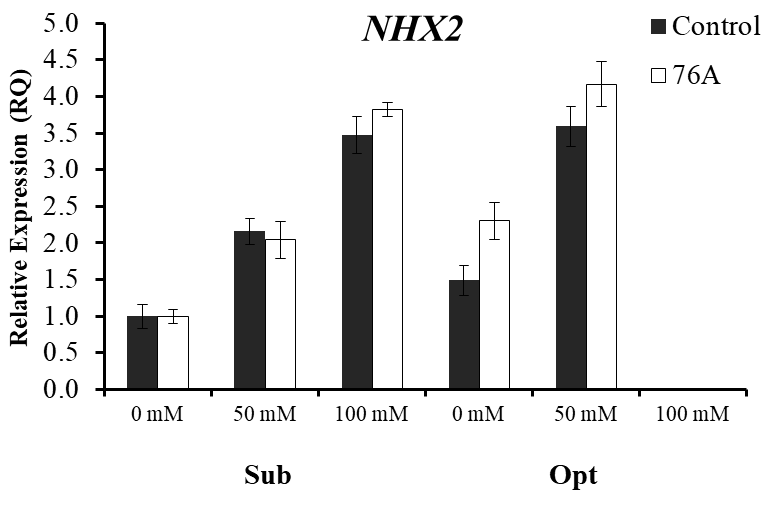


Additional file 1: Figure S1 Gene Expression of ion transporters. (A) NHX1, (B) NHX2, (C) HKT1;1, relative Expression (RQ) quantified by qRT-PCR in samples of uninoculated (control) and inoculated (76A) plants treated with: 0 mM, 50 mM, and 100 mM NaCl. Single asterisks denote significant differences according to Student (P<0.1) between untreated controls and inoculated, double asterisks denote (P<0.05) and triple asterisks denote (P<0.01) between untreated controls and inoculated plants.

Additional file 1: Table S1: Primers used in this study

|  |  |  |
| --- | --- | --- |
|  |  |  |
|  |  |  |
| SlActin-F | Solyc03g078400.2 | TGAATGCACGGTAGCAAACAACAGATT |
| SlActin-R | Solyc03g078400.2 | AATGCATCAGGCACCTCTCAAGTAT |
| SlNii-F | Solyc01g108630.2 | GGATTCATGGGATGCCTGACTAGA |
| SlNii-R-R | Solyc01g108630.2 | TTCTCGTGGAACTGCACCAAAGT |
| SlAMT2-F | Solyc04g050440.2 | TTGTACCGCCGCTCTGACAACTTT |
| SlAMT2-R | Solyc04g050440.2 | CATGGCTCAACAACTGCACAACCT |
| SlNRT2.1-F | Solyc06g074990.1 | TCCAGTCAAGGGAACGGAAGAACA |
| SlNRT2.1-R | Solyc06g074990.1 | ACCACGCTCTGATCGGCAATTT |
| SlNR-F | Solyc01g087550.2 | CAAGCAATCCATCTCCCAT |
| SlNR-R | Solyc01g087550.2 | CATCTCTGTATCGTCTTCAGGA |
| SlLEA-F | Solyc03g116390.2 | AGCGATGCTCCTCACTTGTT |
| SlLEA-R | Solyc03g116390.2 | CGAAGGAGAAGGCTAGTGGA |
| SlSOS1-F  SlSOS1-R | Solyc01g005020  Solyc01g005020 | TCGAGTGATGATTCTGGTGG  ATCACAGTGTGGAAAGGCT |
| SlNHX2-F  SlNHX2-R | Solyc04g056600.2  Solyc04g056600.2 | ATTGGAGGATCGGCAGGAAC  CCATGGAGCCAGATTGACCA |
| SlNHX1-F  SlNHX1-F | Solyc06g008820.2  Solyc06g008820.2 | CACGATATGGTGGGCTGGTT  GGGTGTGGCCAAATCTCGTA |
| SlHKT1.1-F | Solyc07g014680.2 | TCTAGCCCAAGAAACTCAAAT |
| SlHKT1.1-R | Solyc07g014680.2 | CTAATGTTACAACTCCAAGGAATT |
